# Supplementary material for: Counteracting the effects of TNF receptor‐1 has therapeutic potential in Alzheimer's disease
Source: EMBO Mol Med. 2018 Feb 22;10(4):e8300. doi: 10.15252/emmm.201708300 (PMC5887909; doi:10.15252/emmm.201708300)
Supplement: Supplementary file 1 — Appendix [file EMMM-10-e8300-s001.pdf]

Table of contents

Appendix Table S1

Appendix Table S2

Appendix Table S1

Gene	*Forward*	*Reverse*
Il1β	CACCTCACAAGCAGAGCACAAG	GCATTAGAAACAGTCCAGCCCATAC
Il6	TAGTCCTTCCTACCCCAATTTCC	TTGGTCCTTAGCCACTCCTTC
Nos2	CAGCTGGGCTGTACAAACCTT	CATTGGAAGTGAAGCGTTTCG
Tnf	ACCCTGGTATGAGCCCATATAC	ACACCCATTCCCTTCACAGAG
Tnfrsf1a	GCCTCCCGCGATAAAGCCAACC	CTTTGCCCACTTTCACCCACAGG
Tnfrsf1b	ACACCCTACAAACCGGAACC	AGCCTTCCTGTCATAGTATTCCT
Nf-κb	CGTTCCTGCACTTGGCAATCA	GCCAGCTTTCAGAAGTGCCTCA
Lcn2	TGGCCCTGAGTGTCATGTG	CTCTTGTAGCTCATAGATGGTGC
Cxcl9	TCCTTTTGGGCATCATCTTCC	TTTGTAGTGGATCGTGCCTCG
Bace1	AGACGCTACACATCCTGGTG	CCTGGGTGTAGGGCACATAC
Cldn1	TCTACGAGGGACTGTGGATG	TCAGATTCAGCAAGGAGTCG
Cldn5	GCAAGGTGTATGAATCTGTGCT	GTCAAGGTAACAAAGAGTGCCA
Ocln	CCAGGCAGCGTGTTCCT	TTCTAAATAACAGTCACCTGAGGGC
Mmp3	AGTCTACAAGTCCTCCACAG	TTGGTGATGTCTCAGGTTCC
Mmp8	ATTCCCAAGGAGTGTCCAAGC	TGATTGTCATATCTCCAGCACTGG
Mmp9	CTGGACAGCCAGACACTAAAG	CTCGCGGCAAGTCTTCAGAG
Mmp13	TTTATTGTTGCTGCCCATGA	GGTCCTTGGAGTGATCCAGA
Gapdh	TGAAGCAGGCATCTGAGGG	CGAAGGTGGAAGAGTGGGAG
Hprt	AGTGTTGGATACAGGCCAGAC	CGTGATTCAAATCCCTGAAGT
Rpl	CCTGCTGCTCTCAAGGTT	TGGTTGTCACTGCCTGGTACTT
Ubc	AGGTCAAACAGGAAGACAGACGTA	TCACACCCAAGAACAAGCACA
Β-actin	GCTTCTAGGCGGACTGTTACTGA	GCCATGCCAATGTTGTCTCTTAT

Appendix Table S1. Overview of the sequences of the forward and reverse primers used for qPCR analysis

Appendix Table S2

Figure	Panel	Comparison	p-value	n-value
**Figure 2**	**A**	*Il6*<br>APP/PS1$^{wt/wt}$ vs APP/PS1$^{tg/wt}$	0.0271	n=4 and n=3
		Il1β<br>APP/PS1$^{wt/wt}$ vs APP/PS1$^{tg/wt}$	0.0083	n=4 and n=3
		Nos2<br>APP/PS1$^{wt/wt}$ vs APP/PS1$^{tg/wt}$	0.0065	n=4 and n=3
		Nf-κB<br>APP/PS1$^{wt/wt}$ vs APP/PS1$^{tg/wt}$	0.9714	n=4 and n=3
		Cxcl9<br>APP/PS1$^{wt/wt}$ vs APP/PS1$^{tg/wt}$	0.8857	n=4 and n=3
		Lcn2<br>APP/PS1$^{wt/wt}$ vs APP/PS1$^{tg/wt}$	0.0028	n=4 and n=3
		Tnf<br>APP/PS1$^{wt/wt}$ vs APP/PS1$^{tg/wt}$	0.3603	n=4 and n=3
		Tnfrsf1a<br>APP/PS1$^{wt/wt}$ vs APP/PS1$^{tg/wt}$	0.4857	n=4 and n=3
		Tnfrsf1b<br>APP/PS1$^{wt/wt}$ vs APP/PS1$^{tg/wt}$	0.8857	n=4 and n=3
		Mmp3<br>APP/PS1$^{wt/wt}$ vs APP/PS1$^{tg/wt}$	0.1143	n=4 and n=3
		Mmp8<br>APP/PS1$^{wt/wt}$ vs APP/PS1$^{tg/wt}$	0.0496	n=4 and n=3
		Mmp9<br>APP/PS1$^{wt/wt}$ vs APP/PS1$^{tg/wt}$	0.8857	n=4 and n=3
		Mmp13<br>APP/PS1$^{wt/wt}$ vs APP/PS1$^{tg/wt}$	0.0184	n=4 and n=3
	**B**	*Il6*<br>APP/PS1$^{wt/wt}$ vs APP/PS1$^{tg/wt}$	0.7529	n=3 and n=4
		Il1β<br>APP/PS1$^{wt/wt}$ vs APP/PS1$^{tg/wt}$	0.2286	n=3 and n=4
		Nos2<br>APP/PS1$^{wt/wt}$ vs APP/PS1$^{tg/wt}$	0.0286	n=3 and n=4
		Nf-κB<br>APP/PS1$^{wt/wt}$ vs APP/PS1$^{tg/wt}$	0.0286	n=3 and n=4
		Cxcl9<br>APP/PS1$^{wt/wt}$ vs APP/PS1$^{tg/wt}$	0.0123	n=3 and n=3
		Lcn2<br>APP/PS1$^{wt/wt}$ vs APP/PS1$^{tg/wt}$	0.0014	n=3 and n=4
		Tnf<br>APP/PS1$^{wt/wt}$ vs APP/PS1$^{tg/wt}$	0.0056	n=3 and n=3
		Tnfrsf1a<br>APP/PS1$^{wt/wt}$ vs APP/PS1$^{tg/wt}$	0.0446	n=3 and n=3
		Tnfrsf1b<br>APP/PS1$^{wt/wt}$ vs APP/PS1$^{tg/wt}$	0.0658	n=3 and n=3
		Mmp3<br>APP/PS1$^{wt/wt}$ vs APP/PS1$^{tg/wt}$	0.0308	n=3 and n=4
		Mmp8<br>APP/PS1$^{wt/wt}$ vs APP/PS1$^{tg/wt}$	0.0134	n=3 and n=2
		Mmp9<br>APP/PS1$^{wt/wt}$ vs APP/PS1$^{tg/wt}$	0.8571	n=3 and n=4
		Mmp13<br>APP/PS1$^{wt/wt}$ vs APP/PS1$^{tg/wt}$	0.0012	n=3 and n=4
	**C**	*Il6*<br>scrambled vs AβO	<0.0001	n=6 and n=5
		Il1β<br>scrambled vs AβO	<0.0001	n=6 and n=5
		Nos2<br>scrambled vs AβO	0.0006	n=6 and n=7

		Nf-κB<br>scrambled vs AβO	<0.0001	n=6 and n=5
		Cxcl9<br>scrambled vs AβO	<0.0001	n=6 and n=7
		Lcn2<br>scrambled vs AβO	0.0021	n=6 and n=4
		Tnf<br>scrambled vs AβO	0.0002	n=6 and n=7
		Tnfrsf1a<br>scrambled vs AβO	0.0032	n=5 and n=5
		Tnfrsf1b<br>scrambled vs AβO	0.0031	n=4 and n=5
		Mmp3<br>scrambled vs AβO	0.0079	n=5 and n=5
		Mmp8<br>scrambled vs AβO	0.0079	n=5 and n=5
		Mmp9<br>scrambled vs AβO	0.0057	n=6 and n=7
		Mmp13<br>scrambled vs AβO	0.0619	n=4 and n=5
	**D**	*Il6*<br>scrambled vs AβO	0.0040	n=6 and n=7
		Il1β<br>scrambled vs AβO	0.0245	n=6 and n=7
		Nos2<br>scrambled vs AβO	0.0230	n=6 and n=7
		Nf-κB<br>scrambled vs AβO	0.0007	n=6 and n=7
		Cxcl9<br>scrambled vs AβO	<0.0001	n=6 and n=7
		Lcn2<br>scrambled vs AβO	0.0003	n=6 and n=7
		Tnf<br>scrambled vs AβO	0.0116	n=6 and n=7
		Tnfrsf1a<br>scrambled vs AβO	0.1834	n=6 and n=4
		Tnfrsf1b<br>scrambled vs AβO	0.4266	n=6 and n=4
		Mmp3<br>scrambled vs AβO	0.1527	n=4 and n=3
		Mmp8<br>scrambled vs AβO	0.0002	n=5 and n=4
		Mmp9<br>scrambled vs AβO	0.0297	n=6 and n=7
		Mmp13<br>scrambled vs AβO	0.0035	n=4 and n=5
**Figure 3**	**A**	APP/PS1$^{wt/wt}$TNFR1$^{+/+}$ vs<br>APP/PS1$^{tg/wt}$TNFR1$^{+/+}$	0.0271	n=3 and n=3
		APP/PS1$^{wt/wt}$TNFR1$^{+/+}$ vs<br>APP/PS1$^{tg/wt}$TNFR1$^{-/-}$	0.0305	n=3 and n=3
		APP/PS1$^{tg/wt}$TNFR1$^{+/+}$ vs<br>APP/PS1$^{tg/wt}$TNFR1$^{-/-}$	0.0290	n=3 and n=3
	**B**	APP/PS1$^{wt/wt}$TNFR1$^{+/+}$ vs<br>APP/PS1$^{tg/wt}$TNFR1$^{+/+}$	0.0047	n=2 and n=3
		APP/PS1$^{wt/wt}$TNFR1$^{+/+}$ vs<br>APP/PS1$^{tg/wt}$TNFR1$^{-/-}$	0.1953	n=2 and n=3
		APP/PS1$^{tg/wt}$TNFR1$^{+/+}$ vs<br>APP/PS1$^{tg/wt}$TNFR1$^{-/-}$	0.3038	n=3 and n=3
	**C**	APP/PS1$^{wt/wt}$TNFR1$^{+/+}$ vs<br>APP/PS1$^{tg/wt}$TNFR1$^{+/+}$	0.2339	n=4 and n=2
		APP/PS1$^{wt/wt}$TNFR1$^{+/+}$ vs<br>APP/PS1$^{tg/wt}$TNFR1$^{-/-}$	0.0297	n=4 and n=4

		APP/PS1$^{tg/wt}$TNFR1$^{+/+}$ vs APP/PS1$^{tg/wt}$TNFR1$^{-/-}$	0.0496	n=2 and n=4
	D	APP/PS1$^{wt/wt}$TNFR1$^{+/+}$ vs APP/PS1$^{tg/wt}$TNFR1$^{+/+}$	0.0109	n=2 and n=3
		APP/PS1$^{wt/wt}$TNFR1$^{+/+}$ vs APP/PS1$^{tg/wt}$TNFR1$^{-/-}$	0.3761	n=2 and n=2
		APP/PS1$^{tg/wt}$TNFR1$^{+/+}$ vs APP/PS1$^{tg/wt}$TNFR1$^{-/-}$	0.0225	n=3 and n=2
	E	APP/PS1$^{wt/wt}$TNFR1$^{+/+}$ vs APP/PS1$^{tg/wt}$TNFR1$^{+/+}$	0.2270	n=3 and n=4
		APP/PS1$^{wt/wt}$TNFR1$^{+/+}$ vs APP/PS1$^{tg/wt}$TNFR1$^{-/-}$	0.1801	n=3 and n=2
		APP/PS1$^{tg/wt}$TNFR1$^{+/+}$ vs APP/PS1$^{tg/wt}$TNFR1$^{-/-}$	0.6093	n=4 and n=2
	F	APP/PS1$^{wt/wt}$TNFR1$^{+/+}$ vs APP/PS1$^{tg/wt}$TNFR1$^{+/+}$	0.8443	n=3 and n=4
		APP/PS1$^{wt/wt}$TNFR1$^{+/+}$ vs APP/PS1$^{tg/wt}$TNFR1$^{-/-}$	0.8794	n=3 and n=2
		APP/PS1$^{tg/wt}$TNFR1$^{+/+}$ vs APP/PS1$^{tg/wt}$TNFR1$^{-/-}$	0.9758	n=4 and n=2
	G	APP/PS1$^{wt/wt}$TNFR1$^{+/+}$ vs APP/PS1$^{tg/wt}$TNFR1$^{+/+}$	0.0158	n=3 and n=3
		APP/PS1$^{wt/wt}$TNFR1$^{+/+}$ vs APP/PS1$^{tg/wt}$TNFR1$^{-/-}$	0.5594	n=3 and n=2
		APP/PS1$^{tg/wt}$TNFR1$^{+/+}$ vs APP/PS1$^{tg/wt}$TNFR1$^{-/-}$	0.0431	n=3 and n=2
	H	APP/PS1$^{wt/wt}$TNFR1$^{+/+}$ vs APP/PS1$^{tg/wt}$TNFR1$^{+/+}$	0.0056	n=3 and n=3
		APP/PS1$^{wt/wt}$TNFR1$^{+/+}$ vs APP/PS1$^{tg/wt}$TNFR1$^{-/-}$	0.1453	n=3 and n=2
		APP/PS1$^{tg/wt}$TNFR1$^{+/+}$ vs APP/PS1$^{tg/wt}$TNFR1$^{-/-}$	0.0135	n=3 and n=2
	I	TNFR1$^{+/+}$ scrambled vs TNFR1$^{+/+}$ AβO	<0.0001	n=6 and n=5
		TNFR1$^{+/+}$ scrambled vs TNFR1$^{-/-}$ AβO	0.0003	n=6 and n=4
		TNFR1$^{+/+}$ AβO vs TNFR1$^{-/-}$ AβO	0.0196	n=5 and n=4
	J	TNFR1$^{+/+}$ scrambled vs TNFR1$^{+/+}$ AβO	<0.0001	n=6 and n=4
		TNFR1$^{+/+}$ scrambled vs TNFR1$^{-/-}$ AβO	0.0017	n=6 and n=4
		TNFR1$^{+/+}$ AβO vs TNFR1$^{-/-}$ AβO	0.0001	n=4 and n=4
	K	TNFR1$^{+/+}$ scrambled vs TNFR1$^{+/+}$ AβO	<0.0001	n=6 and n=5
		TNFR1$^{+/+}$ scrambled vs TNFR1$^{-/-}$ AβO	0.0011	n=6 and n=4
		TNFR1$^{+/+}$ AβO vs TNFR1$^{-/-}$ AβO	<0.0001	n=5 and n=4
	L	TNFR1$^{+/+}$ scrambled vs TNFR1$^{+/+}$ AβO	<0.0001	n=5 and n=3
		TNFR1$^{+/+}$ scrambled vs TNFR1$^{-/-}$ AβO	<0.0001	n=5 and n=4
		TNFR1$^{+/+}$ AβO vs TNFR1$^{-/-}$ AβO	0.0007	n=3 and n=4
	M	TNFR1$^{+/+}$ scrambled vs TNFR1$^{+/+}$ AβO	0.0317	n=3 and n=2
		TNFR1$^{+/+}$ scrambled vs TNFR1$^{-/-}$ AβO	0.8166	n=3 and n=4
		TNFR1$^{+/+}$ AβO vs TNFR1$^{-/-}$ AβO	0.0019	n=2 and n=4
	N	TNFR1$^{+/+}$ scrambled vs TNFR1$^{+/+}$ AβO	0.0023	n=4 and n=3
		TNFR1$^{+/+}$ scrambled vs TNFR1$^{-/-}$ AβO	0.1311	n=4 and n=5
		TNFR1$^{+/+}$ AβO vs TNFR1$^{-/-}$ AβO	0.0166	n=3 and n=5
	O	TNFR1$^{+/+}$ scrambled vs TNFR1$^{+/+}$ AβO	0.0014	n=5 and n=4
		TNFR1$^{+/+}$ scrambled vs TNFR1$^{-/-}$ AβO	0.2745	n=5 and n=4
		TNFR1$^{+/+}$ AβO vs TNFR1$^{-/-}$ AβO	0.0083	n=4 and n=4
	P	TNFR1$^{+/+}$ scrambled vs TNFR1$^{+/+}$ AβO	0.3034	n=5 and n=3
		TNFR1$^{+/+}$ scrambled vs TNFR1$^{-/-}$ AβO	0.2380	n=5 and n=4
		TNFR1$^{+/+}$ AβO vs TNFR1$^{-/-}$ AβO	0.0261	n=3 and n=4
**Figure 6**	A	TNFR1$^{+/+}$ scrambled vs TNFR1$^{+/+}$ AβO	<0.0001	n=13 and n=12
		TNFR1$^{+/+}$ scrambled vs TNFR1$^{-/-}$ AβO	0.6966	n=13 and n=12
		TNFR1$^{+/+}$ AβO vs TNFR1$^{-/-}$ AβO	<0.0001	n=12 and n=12

	**B**	$TNFR1^{+/+}$ scrambled vs $TNFR1^{+/+}$ AβO	<0.0001	n=4 and n=6
		$TNFR1^{+/+}$ scrambled vs $TNFR1^{-/-}$ AβO	0.0068	n=4 and n=6
		$TNFR1^{+/+}$ AβO vs $TNFR1^{-/-}$ AβO	0.0096	n=6 and n=6
	**C**	$TNFR1^{+/+}$ scrambled vs $TNFR1^{+/+}$ AβO	0.0010	n=4 and n=6
		$TNFR1^{+/+}$ scrambled vs $TNFR1^{-/-}$ AβO	0.0259	n=4 and n=6
		$TNFR1^{+/+}$ AβO vs $TNFR1^{-/-}$ AβO	<0.0001	n=6 and n = 6
	**D**	$TNFR1^{+/+}$ scrambled vs $TNFR1^{+/+}$ AβO	0.0027	n=4 and n=6
		$TNFR1^{+/+}$ scrambled vs $TNFR1^{-/-}$ AβO	0.9665	n=4 and n=6
		$TNFR1^{+/+}$ AβO vs $TNFR1^{-/-}$ AβO	0.0039	n=6 and n=6
	**E**	$TNFR1^{+/+}$ scrambled vs $TNFR1^{+/+}$ AβO	0.0025	n=4 and n=6
		$TNFR1^{+/+}$ scrambled vs $TNFR1^{-/-}$ AβO	0.1597	n=4 and n=6
		$TNFR1^{+/+}$ AβO vs $TNFR1^{-/-}$ AβO	0.0030	n=6 and n =6
**Figure 7**	**A**	$APP/PS1^{tg/wt}TNFR1^{+/+}$ vs $APP/PS1^{tg/wt}TNFR1^{-/-}$	0.0258	n=4 and n=6
	**B**	≤ 10 μm^2 $APP/PS1^{tg/wt}TNFR1^{+/+}$ vs ≤ 10 μm^2 $APP/PS1^{tg/wt}TNFR1^{-/-}$	0.0396	n=4 and n=6
		> 10 μm^2; ≤ 20 μm^2 $APP/PS1^{tg/wt}TNFR1^{+/+}$ vs > 10 μm^2; ≤ 20 μm^2 $APP/PS1^{tg/wt}TNFR1^{-/-}$	0.4176	n=4 and n=6
		> 20 μm^2 $APP/PS1^{tg/wt}TNFR1^{+/+}$ vs > 20 μm^2 $APP/PS1^{tg/wt}TNFR1^{-/-}$	0.9959	n=4 and n=6
	**E**	$APP/PS1^{wt/wt}TNFR1^{+/+}$ vs $APP/PS1^{tg/wt}TNFR1^{+/+}$	<0.0001	n=3 and n=6
		$APP/PS1^{wt/wt}TNFR1^{+/+}$ vs $APP/PS1^{tg/wt}TNFR1^{-/-}$	<0.0001	n=3 and n=9
		$APP/PS1^{tg/wt}TNFR1^{+/+}$ vs $APP/PS1^{tg/wt}TNFR1^{-/-}$	0.3467	n=6 and n=9
	**F**	$APP/PS1^{wt/wt}TNFR1^{+/+}$ vs $APP/PS1^{tg/wt}TNFR1^{+/+}$	<0.0001	n=3 and n=6
		$APP/PS1^{wt/wt}TNFR1^{+/+}$ vs $APP/PS1^{tg/wt}TNFR1^{-/-}$	<0.0001	n=3 and n=9
		$APP/PS1^{tg/wt}TNFR1^{+/+}$ vs $APP/PS1^{tg/wt}TNFR1^{-/-}$	0.0255	n=6 and n=9
	**G**	$APP/PS1^{wt/wt}TNFR1^{+/+}$ vs $APP/PS1^{tg/wt}TNFR1^{+/+}$	0.0003	n=2 and n=6
		$APP/PS1^{wt/wt}TNFR1^{+/+}$ vs $APP/PS1^{tg/wt}TNFR1^{-/-}$	0.0003	n=2 and n=9
		$APP/PS1^{tg/wt}TNFR1^{+/+}$ vs $APP/PS1^{tg/wt}TNFR1^{-/-}$	0.9102	n=6 and n=9
	**H**	$APP/PS1^{wt/wt}TNFR1^{+/+}$ vs $APP/PS1^{tg/wt}TNFR1^{+/+}$	<0.0001	n=3 and n=6
		$APP/PS1^{wt/wt}TNFR1^{+/+}$ vs $APP/PS1^{tg/wt}TNFR1^{-/-}$	<0.0001	n=3 and n=8
		$APP/PS1^{tg/wt}TNFR1^{+/+}$ vs $APP/PS1^{tg/wt}TNFR1^{-/-}$	0.0775	n=6 and n=8
	**I**	$APP/PS1^{wt/wt}TNFR1^{+/+}$ vs $APP/PS1^{tg/wt}TNFR1^{+/+}$	<0.0001	n=4 and n=3
		$APP/PS1^{wt/wt}TNFR1^{+/+}$ vs $APP/PS1^{tg/wt}TNFR1^{-/-}$	0.5290	n=4 and n=4
		$APP/PS1^{tg/wt}TNFR1^{+/+}$ vs $APP/PS1^{tg/wt}TNFR1^{-/-}$	<0.0001	n=3 and n=4
**Figure 8**	**B**	$APP/PS1^{wt/wt}TNFR1^{+/+}$ vs $APP/PS1^{tg/wt}TNFR1^{+/+}$	0.0088	n=3 and n=4
		$APP/PS1^{wt/wt}TNFR1^{+/+}$ vs $APP/PS1^{tg/wt}TNFR1^{-/-}$	0.2308	n=3 and n=6
		$APP/PS1^{tg/wt}TNFR1^{+/+}$ vs $APP/PS1^{tg/wt}TNFR1^{-/-}$	0.0659	n=4 and n=6

	D	APP/PS1$^{wt/wt}$TNFR1$^{+/+}$ vs APP/PS1$^{tg/wt}$TNFR1$^{+/+}$	0.0156	n=2 and n=2
		APP/PS1$^{wt/wt}$TNFR1$^{+/+}$ vs APP/PS1$^{tg/wt}$TNFR1$^{-/-}$	0.5878	n=2 and n=3
		APP/PS1$^{tg/wt}$TNFR1$^{+/+}$ vs APP/PS1$^{tg/wt}$TNFR1$^{-/-}$	0.0235	n=2 and n=3
**Figure 9**	A	APP/PS1$^{wt/wt}$TNFR1$^{+/+}$ vs APP/PS1$^{tg/wt}$TNFR1$^{+/+}$	<0.0001	n=19 and n=10
		APP/PS1$^{wt/wt}$TNFR1$^{+/+}$ vs APP/PS1$^{tg/wt}$TNFR1$^{-/-}$	0.0356	n=19 and n=11
		APP/PS1$^{tg/wt}$TNFR1$^{+/+}$ vs APP/PS1$^{tg/wt}$TNFR1$^{-/-}$	0.1001	n=10 and n=11
	B	APP/PS1$^{wt/wt}$TNFR1$^{+/+}$ vs APP/PS1$^{tg/wt}$TNFR1$^{+/+}$	0.0231	n=18 and n=8
		APP/PS1$^{wt/wt}$TNFR1$^{+/+}$ vs APP/PS1$^{tg/wt}$TNFR1$^{-/-}$	0.7677	n=18 and n=10
		APP/PS1$^{tg/wt}$TNFR1$^{+/+}$ vs APP/PS1$^{tg/wt}$TNFR1$^{-/-}$	0.0114	n=8 and n=10
	C	TNFR1$^{+/+}$ scrambled vs TNFR1$^{+/+}$ AβO	0.0031	n=11 and n=6
		TNFR1$^{+/+}$ scrambled vs TNFR1$^{-/-}$ AβO	0.3159	n=11 and n=13
		TNFR1$^{+/+}$ AβO vs TNFR1$^{-/-}$ AβO	0.0461	n=6 and n=13
	D	TNFR1$^{+/+}$ scrambled vs TNFR1$^{+/+}$ AβO	0.0063	n=11 and n=6
		TNFR1$^{+/+}$ scrambled vs TNFR1$^{-/-}$ AβO	0.5905	n=11 and n=11
		TNFR1$^{+/+}$ AβO vs TNFR1$^{-/-}$ AβO	0.0428	n=6 and n=11
	E	PBS scrambled vs PBS AβO	0.0329	n=6 and n=9
		PBS scrambled vs TROS AβO	0.7959	n=6 and n=6
		PBS AβO vs TROS AβO	0.0073	n=9 and n=6
	F	PBS scrambled vs PBS AβO	0.0017	n=5 and n=9
		PBS scrambled vs TROS AβO	0.0529	n=5 and n=8
		PBS AβO vs TROS AβO	0.2129	n=9 and n=8
**Figure EV3**	A	WT scrambled/PBS vs WT AβO/PBS	0.0015	n=6 and n=6
		WT scrambled/PBS vs WT AβO/TROS	0.0057	n=6 and n=6
		WT AβO/PBS vs WT AβO/TROS	0.587	n=6 and n=6
	B	WT scrambled/PBS vs WT AβO/PBS	<0.0001	n=6 and n=6
		WT scrambled/PBS vs WT AβO/TROS	<0.0001	n=6 and n=6
		WT AβO/PBS vs WT AβO/TROS	0.5276	n=6 and n=6
	C	WT scrambled/PBS vs WT AβO/PBS	0.0005	n=6 and n=6
		WT scrambled/PBS vs WT AβO/TROS	0.0004	n=6 and n=6
		WT AβO/PBS vs WT AβO/TROS	0.8243	n=6 and n=6
	D	WT scrambled/PBS vs WT AβO/PBS	<0.0001	n=6 and n=6
		WT scrambled/PBS vs WT AβO/TROS	0.0015	n=6 and n=6
		WT AβO/PBS vs WT AβO/TROS	0.3449	n=6 and n=6
	E	WT scrambled/PBS vs WT AβO/PBS	<0.0001	n=6 and n=6
		WT scrambled/PBS vs WT AβO/TROS	<0.0001	n=6 and n=6
		WT AβO/PBS vs WT AβO/TROS	0.5947	n=6 and n=6

Appendix Table S2. Overview of exact p- and n-values of all figures
