# Supplementary material for: Counteracting the effects of TNF receptor‐1 has therapeutic potential in Alzheimer's disease
Source: EMBO Mol Med. 2018 Feb 22;10(4):e8300. doi: 10.15252/emmm.201708300 (PMC5887909; doi:10.15252/emmm.201708300)
Supplement: Supplementary file 2 — Expanded View Figures PDF [file EMMM-10-e8300-s002.pdf]

Expanded View Figures

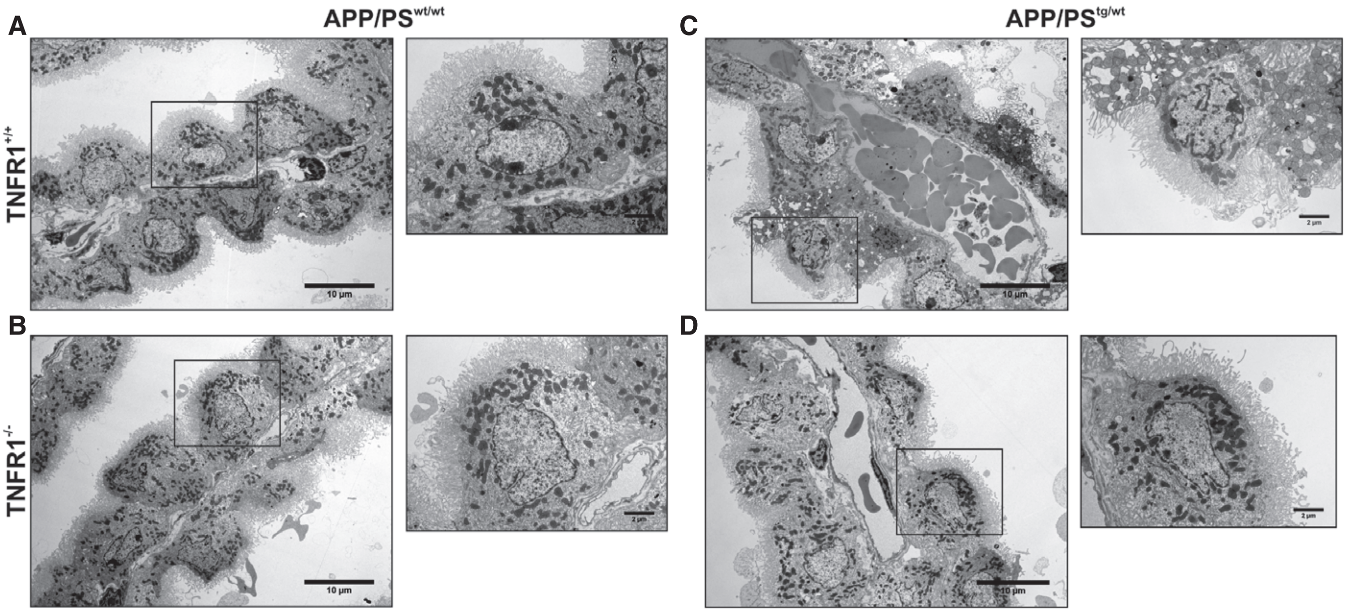

Figure EV1. TNFR1 deficiency protects against morphological alterations in the choroid plexus of APP/PS1$^{tg/wt}$ mice determined by TEM.

A–D Representative conventional transmission electron microscopy (TEM) images of the choroid plexus of 18-week-old C57BL/6J APP/PS1$^{tg/wt}$ mice in a TNFR1$^{+/+}$ and TNFR1$^{-/-}$ background compared to age-matched non-transgenic controls. In non-transgenic controls (A, B), the cuboidal structure of the choroid plexus epithelial (CPE) cells is maintained. The nuclei have regular shapes and mitochondria look normal (*zoom*). The loss of the cuboidal shape is more enhanced in CPE cells of APP/PS1$^{tg/wt}$ TNFR1$^{+/+}$ mice (C). The capillaries are swollen and filled with plenty of red blood cells, the nuclei have irregular shapes, and some CPE cells are at a degenerative state (*zoom*). In contrast (D), CPE cells of APP/PS1$^{tg/wt}$TNFR1$^{-/-}$ mice have the same cellular shape as non-transgenic littermates. The capillaries are less swollen, and the mitochondria and nuclear shape are normal (*zoom*). The TEM images were taken at a magnification of 1,000×, scale bar represents 10 µm; zooms were taken at a magnification of 3,000×, scale bar represents 2 µm.

© 2018 The Authors

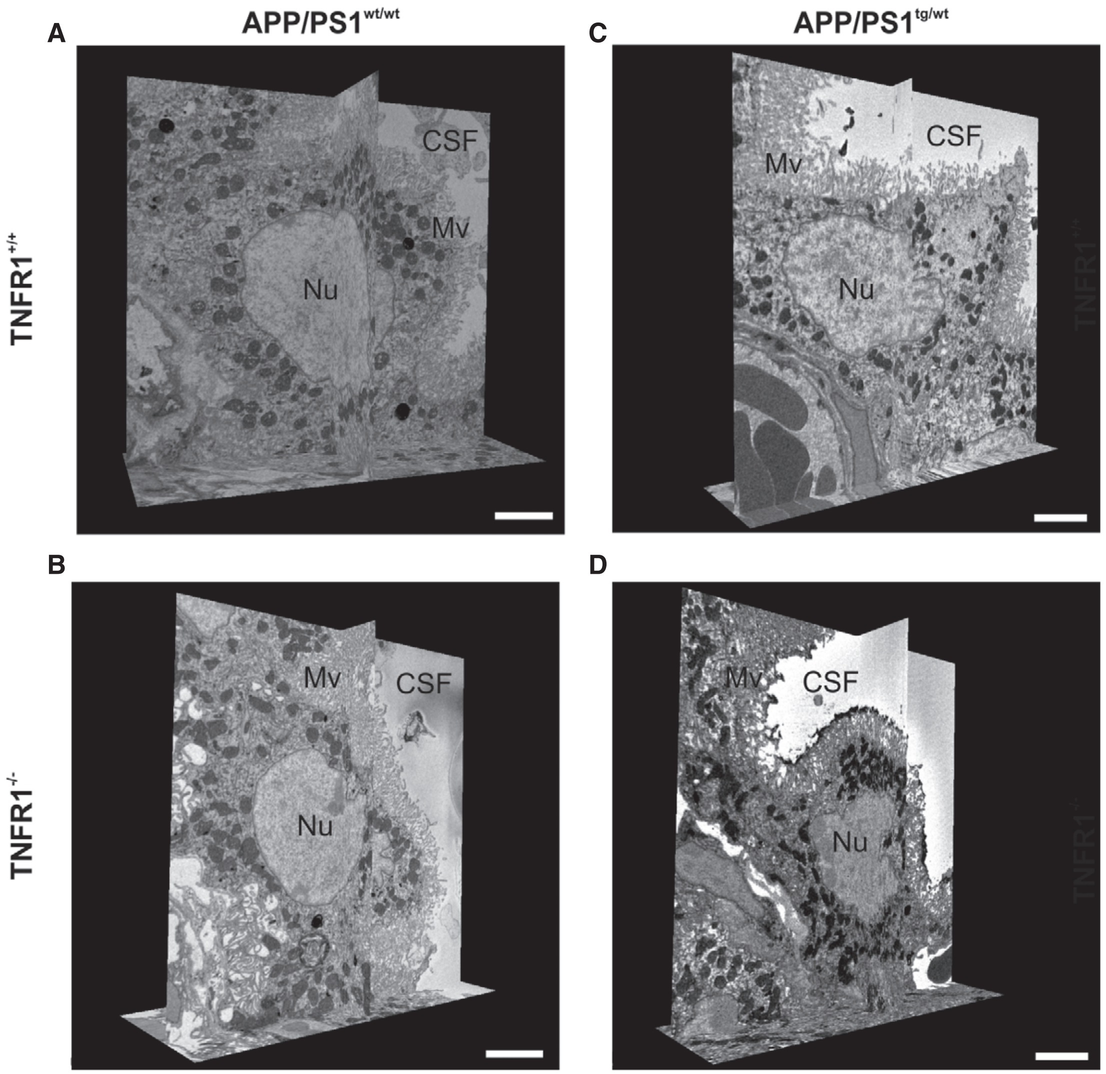

Figure EV2. TNFR1 deficiency protects morphological alterations in the choroid plexus of APP/PS1$^{tg/wt}$ mice determined by SBF-SEM.

A–D Representative serial block-face scanning electron microscopy (SBF-SEM) images of the choroid plexus of 18-week-old C57BL/6J APP/PS1$^{tg/wt}$ mice in a TNFR1$^{+/+}$ (A, C) and TNFR1$^{-/-}$ (B, D) background compared to age-matched non-transgenic controls (*n* = 1/group), derived from Movies EV5–EV8. CSF, cerebrospinal fluid; Mv, microvilli; Nu, nucleus. Scale bar, 5 μm.

 © 2018 The Authors

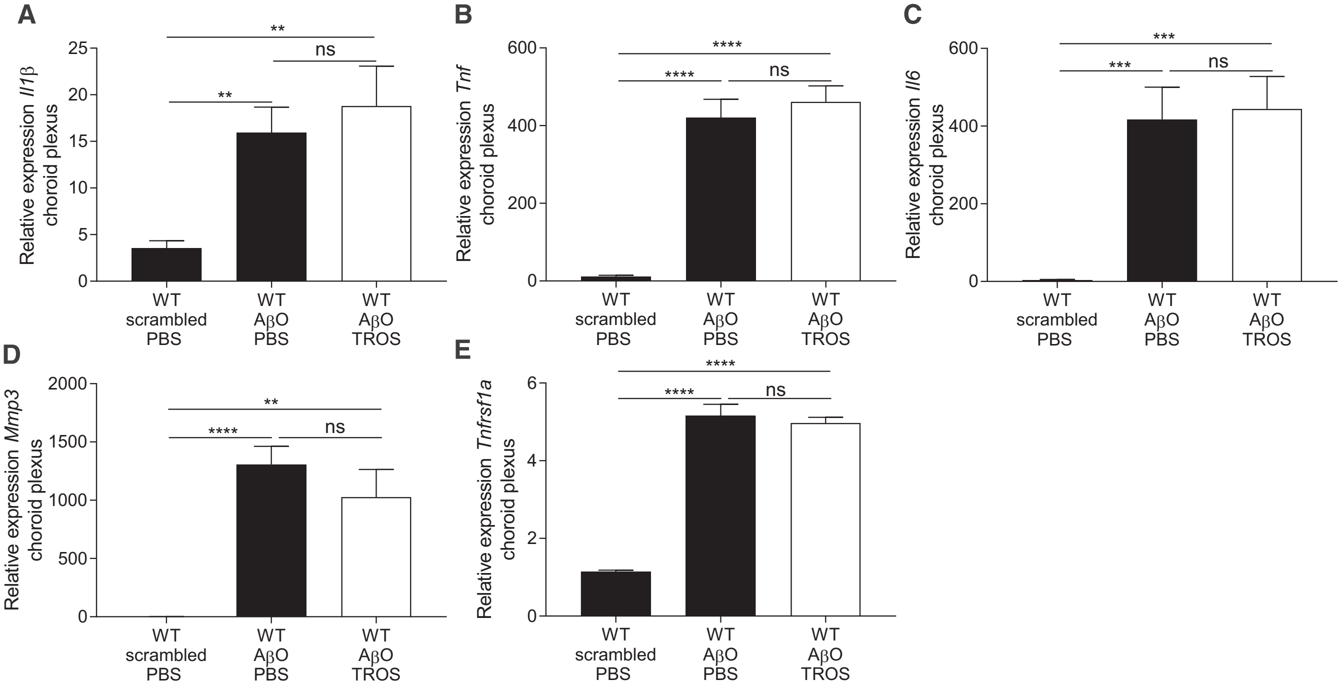

Figure EV3. Icv injection of TROS in WT mice does not affect inflammation induced by $A\beta_{1-42}$ oligomers (AβO).

A–E Relative mRNA gene expression of *Il1β*, *Tnf*, *Il6*, *Mmp3*, and *Tnfrsf1a* of C57BL/6J wild-type (WT) mice 6 h after intracerebroventricular (icv) injection with scrambled peptide or with AβO (1 µg/ml) together with PBS or with TROS 1.55 µg/µl (n = 6/group). Bars represent mean ± SEM. qPCR was normalized to stable housekeeping genes determined by GeNorm. Statistics were performed with an unpaired t-test, **$0.001 \leq P < 0.01$; ***$0.001 \leq P < 0.0001$; ****$P < 0.0001$.

© 2018 The Authors
