# Supplementary material for: Counteracting the effects of TNF receptor‐1 has therapeutic potential in Alzheimer's disease
Source: EMBO Mol Med. 2018 Feb 22;10(4):e8300. doi: 10.15252/emmm.201708300 (PMC5887909; doi:10.15252/emmm.201708300)
Supplement: Supplementary file 6 — Movie EV4 [file EMMM-10-e8300-s006.zip › Movie_EV4_legend.rtf]

Movie EV4:Morphology of choroid plexus epithelial cells of TNFR1-/- mice after injection of Aﬂ-42 oligomers (Aﬂ) into the cerebral ventricles determined by SBF-SEMShown are the representative 3D reconstructions of the choroid plexus from C57BL/6J TNFR1-/- mice 6 h after injection of Aﬂ into the cerebral ventricles, determined by serial block-face scanning electron microscopy (SBF-SEM). 
