# Supplementary material for: Counteracting the effects of TNF receptor‐1 has therapeutic potential in Alzheimer's disease
Source: EMBO Mol Med. 2018 Feb 22;10(4):e8300. doi: 10.15252/emmm.201708300 (PMC5887909; doi:10.15252/emmm.201708300)
Supplement: Supplementary file 10 — Movie EV8 [file EMMM-10-e8300-s010.zip › Movie_EV8_legend.rtf]

Movie EV8:Morphology of choroid plexus epithelial cells of APP/PS1tg/wt mice in a TNFR1-/- background determined by SBF-SEMShown are the representative 3D reconstructions of the choroid plexus from 18 week old C57BL/6J APP/PS1tg/wtTNFR1-/- mice determined by serial block-face scanning electron microscopy (SBF-SEM).
